# Enteral Ca-Intake May Be Low and Affects Serum-PTH-Levels in Pre-school Children With Chronic Kidney Disease

**DOI:** 10.3389/fped.2021.666101

**Published:** 2021-07-20

**Authors:** Lilith Schmitz, Pamela Hoermann, Birgit Trutnau, Augustina Jankauskiene, Ariane Zaloszyc, Alberto Carlo Edefonti, Claus Peter Schmitt, Guenter Klaus

**Affiliations:** ^1^Department of Pediatric Nephrology, University of Marburg, Marburg, Germany; ^2^KfH Pediatric Kidney Center and University Hospital, University of Marburg, Marburg, Germany; ^3^Pediatric Center, Vilnius University, Vilnius, Lithuania; ^4^Division of Pediatric Nephrology, University Hospital Strassbourg, Strassbourg, France; ^5^Pediatric Nephrology, Dialysis and Transplant Unit, Fondazione Cà Granda, Ospedale Policlinico, Milan, Italy; ^6^Division of Pediatric Nephrology, University Hospital Heidelberg, Heidelberg, Germany

**Keywords:** Ca-intake, Ca deficiency, CKD, phosphate binder, children, pre-school age

## Abstract

Treatment of chronic kidney disease (CKD) mineral bone disorder (MBD) is challenging in growing children due to the high amount of calcium needed for normal bone mineralization and the required dietary phosphate restriction, which often includes intake of calcium-rich products such as milk. Therefore, enteral calcium-intake (Ca-I) was calculated.

**Patients:** We looked at pediatric CKD-Patients aged 0–6 years.

**Design:** We used a retrospective analysis of Ca-I from dietary data collections. Ca-I below 60% or above 100% of the D-A-CH and the KDOQI reference values were considered as severe Ca deficiency or Ca overload, respectively.

**Results:** We had 41 children, median age 1.1 (range 0-5.8) years, body weight 7.3 (2.4–19.9) kg, and length 68 (48-105) cm at the time of first dietary data collection. Renal function was classified as CKD stage III in 20, IV in 28, V in 44, and VD in 142 dietary data collections. At the first dietary data collection, 5 children were in the CKD stage III, 10 in IV, 9 in V, and 17 were on dialysis. Only one child progressed to a higher CKD stage. In total, 234 dietary data collections were analyzed, and 65 follow-up collections were available from 33 children after a time interval of 26 (1–372) days. The median caloric intake was 120 (47–217)% of D-A-CH RDI. In 149 (63.6%) of the dietary data collections, enteral Ca-I was below the target (<100% of the D-A-CH and KDOQI RDI). Severe Ca-deficiency was found in 11 (26%) and 4 (12%) of the children at the first and second dietary data collection, respectively. In total, 11 children were on Ca-containing phosphate binders. In dietary data collection 1 and 2, there were seven children. From these, 4/7 and 4/7 patients had an enteral total Ca-I above the 100% D-A-CH-limit or above the KDOQI limit, respectively. Absolute dietary Ca-I and Ca-I normalized to body weight correlated negatively with PTH (r = −0.196, *p* < 0.005 and r = −0.13, *p* < 0.05).

**Conclusion:** Enteral Ca-I should repeatedly be monitored in CKD children because many may may otherwise be underexposed to enteral calcium and overexposed when calcium-containing phosphate binders are given. Our findings suggest a major impact of dietary calcium supply on bone health in pediatric CKD.

## Introduction

In children with chronic kidney disease (CKD), prevention of mineral and bone disorder is essential to prevent skeletal deformities, bone pain, growth retardation, and, most importantly, uremic vascular calcifications. The latter is an important cause of morbidity and mortality in the long term (1, 2). Severe hyperparathyroidism with PTH levels above 500 ng/ml was shown to be associated with bone pain, fractures, or radiological signs of uremic bone disease in pediatric peritoneal dialysis patients (2).

Children with CKD need an age-adapted caloric intake. An adequate caloric food intake including milk products in infants and pre-school children carries the risk of hyperphosphatemia, which is usually noticed from early CKD stages (3). For treatment of hyperphosphatemia, dietary counseling and calcium-containing phosphate binders (CaPB), i.e., calcium carbonate or calcium acetate, are often used (4). High doses of CaPB may result in a positive calcium balance with the risk of extra-osseous calcifications. Therefore, an upper limit of enteral calcium intake was set by the KDOQI nutrition guidelines (5). However, these limits are not supported by clinical studies. The amount of calcium from CaPB absorbed is variable and is estimated to be ~30% of total calcium intake. This proportion is also dependent on the Vitamin D therapy of the patients (5).

Reduction of phosphate intake also decreases calcium intake. In younger children, most calcium is ingested from milk products (6), which are limited or replaced by formula milk, which has a higher calcium content relative to human milk but lower than that of cow‘s milk. The absorption from breast milk is higher compared to formula milk (7). Calcium intake can be reduced by the use of lower calcium formula or a restricted diet. Recently, it was shown, that phosphate and Ca intake are related in pediatric CKD 4-5D patients (8).

A restricted Ca-I has to be balanced against the needs of the children, who must have a positive calcium balance to maintain mineralization of the growing skeleton (9). A significant proportion of pediatric patients have reduced serum calcium levels (2), which results in more severe hyperparathyroidism and are associated with mineralization defects, demonstrated in 26% of children with CKD 2 and up to 80% in CKD 4/5 (10) or in PD patients (11). Consequently, the diet of children with CKD is recommended to be regularly assessed for calcium (12).

This study addresses the question of whether the current calcium intake of children with CKD, aged 0–6 years, is in accordance with the recommended nutritional guidelines. As a reference for the recommended dietary intake of calcium, the German-Austrian-Swiss (D-A-CH)- tables (12) and/or the KDOQI nutrition guidelines (5) were used. To our knowledge, there are no published studies, which investigated the lower limit of Ca-I in pediatric CKD patients, which worsen the secondary hyperparathyroidism or result in mineralization defects of the bone. Therefore, a Ca-I below 100% was defined as Ca deficiency and below 60% of the RDI as severe Ca deficiency. For enteral Ca overload, 100% of the D-A-CH reference and 100% of the KDOQI nutrition guidelines (5) were used.

## Patients and Methods

There were 41 patients with CKD Stage 3 to 5D (Table 1) in the age range from 0–6 years that were recruited from the pediatric nephrology units in Heidelberg (*n* = 23), Marburg (*n* = 12), Milan (*n* = 3), and Strasbourg (*n* = 3). All members of the European Pediatric Dialysis Working group were invited to participate in the study. In the first dietary data collection, 5 children were in the CKD stage III, 10 in IV, 9 in V, and 17 were on dialysis. Only one child progressed to a higher CKD stage during the observation period. Underlying renal disease as typical for this age population with all patients demonstrating congenital anomalies of the kidney and urinary tract (CAKUT) except for two patients (severe bilateral renal vein and inferior vena cava thrombosis and with fetofetal transfusion syndrome). Supplemental cholecalciferol was prescribed in 37 patients and active Vitamin D metabolites in 75 data collections.

**Table 1:** Clinical and biochemical characteristics of the patients.

	**All dietary data collections**	**Dietary data collection 1**	**Dietary data collection 2**
*N* =	41 (14 female)	41 (14 female)	33 (12 female)
Age (y)	1.1 (0-5.8)	0.8 (0.1-5.8)	0.9 (0.1-5.8)
Height (cm)	69 (48-105)	69 (48-105)	63.2 (48-105)
Height-z-score	−2.32 (−4.57-3.53)	−1.56 (−4.57-2.07)	−2.09(−4.57-2.08)
Weight-SDS	−1.75 (−4.94-2.98)	−1.61 (−4.94-2.08)	−1.64(−4.07- −1.73)
CKD stage III/IV/V/VD/not classified	-	5/10/9/170	3/6/6/171
S-Ca (mmol/l)	2.4 (2.0-2.89)	2.43 (2.00-2.89)	2.46 (2.07-2.80)
S-P (mmol/l)	1.4 (0.50-3.5)	1.48 (0.60-2.90)	1.32 (0.80-2.0)
S-PTH (ng/ml)	52 (0.1-2651)	21.0 (0.90-308)	18.2 (0.70-513)
25(OH) vitamin D (nmol/l)	38.7 (12.9-52.7)	na	na

*Values are given as median (range), Measurements of 25(OH); vitamin D, n = 25; na, not applicable*.

Renal function was classified as CKD stage III in 20, IV in 28, V in 44, and VD in 142 dietary data collections.

### Design

It is the policy of the involved centers that children with CKD on enteral feeding obtain an initial dietary analysis and in case of issues regarding weight gain, in case CKD MBD targets repeatedly are not achieved (regarding serum calcium, phosphate and/or PTH), and in case history suggests dietary issues. The available complete 1-day dietary data collections from 2016–2019 in the participating centers were identified retrospectively. The dietary data collections were numbered in chronological order (data collection 1, 2, …). For the present study, only dietary data collections from patients 0–6 years of age were analyzed. The questionnaire (see Supplementary Material) with the food details were sent to the participating centers where they were filled in with the clinical data of the patients as well as the amount and type of food taken. The intake of calories, calcium, and phosphate was calculated centrally (Marburg) using the Prodi 6 software (Fa nutriscience GmbH, Freiburg, Germany). In patients taking CaPB, the amount of elemental calcium from these drugs was also calculated (Calcium carbonate: 40%, Calcium acetate: 25% of weight). The intake calculated from the diary data collections was compared with the daily recommended intake (DRI) according to the actual German-Austrian-Swiss (D-A-CH) tables (13), which are used commonly in central Europe. A calcium intake below 100% was defined as Ca deficiency, below 60% was defined as severe Ca deficiency, and intake above 100% of the D-A-CH reference was defined as Ca overload. In patients taking CaPB, the KDOQI reference (5) was also used with Ca overloud defined as > 100% the upper recommended limit. In addition to the dietary data collections, standard biochemical laboratory results were collected: Serum-calcium (S-Ca), serum-phosphate (S-Phos), creatinine, parathyroid hormone (PTH), 25(OH) Vitamin D, and 25(OH) Vitamin D levels were available in 25 patients only.

The study was approved by the ethics committee of the Philipp‘s University of Marburg (Reference 84/16).

### Statistics

Descriptive data analysis was performed for basic clinical and nutritional parameters and is given as the median and range. For further statistical analysis data were tested for normal distribution. For correlation analysis, Pearson's coefficient was calculated. The statistical significance was set for *p* < 0.05.

## Results

### Dietary Data Collection

Dietary data collections could be analyzed in 41 (14 female) children with CKD from four centers [median age at the first dietary data collection was 0.8 (0-5.8) years, median weight 7.4 (2.4-19.9) kg, and median height 68 (48-105) cm] (Table 1). A total of 234 dietary data collections were obtained. The number of collections per patient varied with 41 patients having one dietary data collection, 33 patients two dietary data collections, and 26 patients having three to six dietary data collections. In three patients, no data were available on the feeding route. Oral feeding was used in 148 dietary data collections and tube feeding either by PEG or nasogastric tube in 55 dietary data collections. Median caloric intake was 120 (56–203)% according to the RDI (D-A-CH). There was no major change during the follow-up dietary data collections during the observation period [122 (63-217)% and 128 (94-175)% for dietary data collection 1 and 2, respectively].

### Ca-Intake

Calculated dietary Ca-Intake was 328 (94-917) mg in all dietary data collection, 296 (94–131) mg in collection 1, and 319 (175–629) mg in collection 2. This corresponded to 93 (23–197)% and 94 (26–198)% of the recommended amount of the D-A-CH reference (13) for the first two evaluations, respectively, A low Ca-I (<100% of D-A-CH RDI) was found in 149/234 (63.6%) dietary data collections. In 11 (26%) and 4 (12%) patients, the dietary Ca-Intake was below 60% of the D-A-CH reference in dietary data collection 1 and 2, respectively. A Ca-Intake above the D-A-CH upper limit was found in 36.2% of all dietary data collections (Table 2).

**Table 2:** Nutrition data collection.

	**All dietary data collections**	**Dietary data collection 1**	**Dietary data collection 2**
*N* =	234	41	33
Calories (% of D-A-CH)	120 (47-217)	122 (63-217)	128 (94-175)
Calcium (mg)	328 (94-917)	296 (94-917)	319 (131-629)
Calcium (% D-A-CH (Median)	92 (47-217)	93 (63-217)	94 (94-175)
Calcium <100% D-A-CH	149 (63.6%)	27 (65.8%)	17 (51.5%)
Calcium <60% D-A-CH	44 (18.8%)	11 (27%)	4 (12%)
Calcium > 100% D-A-CH	85 (36.2%)	13 (31.7%)	13 (39.4%)
Calcium > 100% KDOQI	10 (4.2%)	4 (9.7%)	0

*Values are given as median (range)*.

In total, 11 children were on CaPB or were being treated with CaPB. In those, the total enteral Ca-I was 669 (252–1757) mg, which corresponded to 136 (34–192)% and 86 (36–204)% of the RDI according to D-A-CH (13) and KDOQI (5), respectively. The Ca-I exceeded the 100% of D-A-CH reference as well as the KDOQI upper limit in 21 (72%) and 5 (17%) dietary data collections, respectively. When analyzing the first two dietary data collections only, only seven children took CaPB. From these, 4/7 and 4 /7 patients had an enteral total Ca-I above the 100% D-A-CH-limit or above the KDOQI limit, respectively (Table 3).

**Table 3:** Calcium-Intake in patients taking calcium-containing phosphate binders.

	**All dietary data collections**	**Dietary data collection 1**	**Dietary data collection 2**
*N* =	29	7	2
Calcium (mg)	669 (414-1757)	669 (414-1757)	518(432-605)
Calcium (% D-A-CH (Median)	137 (34-192)	112 (87-154)	88 (36-204)
Calcium <100% D-A-CH (*n*)	8 (28%)	3 (43%)	0
Calcium >100% D-A-CH (*n*)	21 (72%)	4 (57%)	0
Calcium > KDOQI (*n*)	5 (17%)	4 (57%)	0

*Values are given as median (range) or numbers*.

### Effects of Ca-Intake on Biochemical Parameters of CKD-MBD

Serum calcium was within the normal range in most of the measurements. Low total serum calcium (not corrected for albumin) was measured in 13 patients at various time points. PTH was above the recommended target range for CKD stage 5 and pediatric peritoneal dialysis patients (1–3 times the upper limit of normal, i.e., >210 pmol/l) (14, 15) in 40 measurements. Corresponding measurements of 25(OH) Vitamin D were available in 25 patients with a median of 38.7 (12.9–52.7) nmol/l.

Calcium intake (absolute or weight adjusted) did not correlate with S-Ca or S-Phos. In the patients taking CaPB, higher S-Phos was associated with higher Ca-I (r = 0.39, *p* < 0.033).

**Figure 1:**
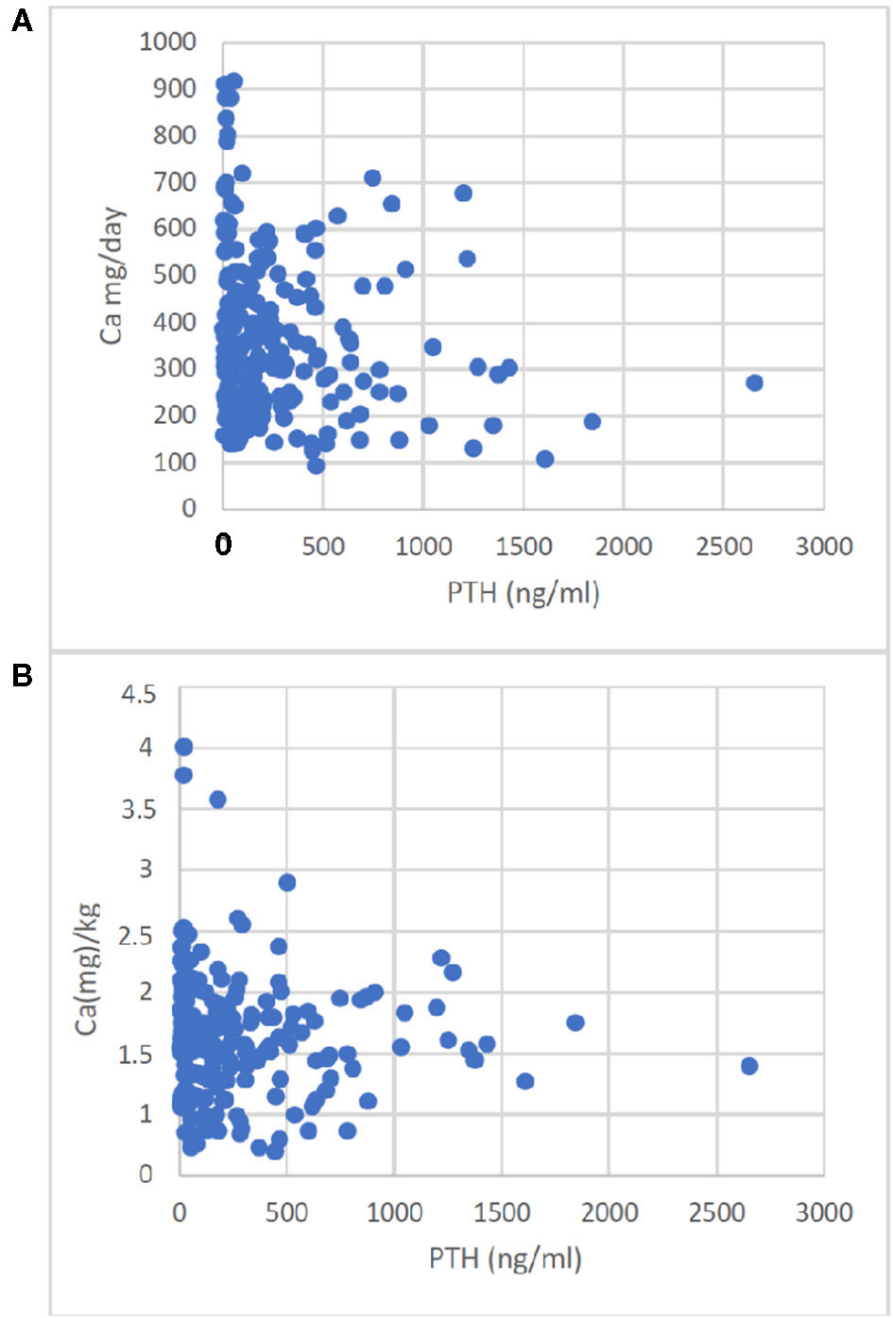
Correlation of Ca-Intake with PTH. Enteral Ca-Intake given as absolute intake **(A)** or given as Ca(mg)/kg body weight **(B)** correlated with PTH. The correlations were A: r = −0.196, *p* < 0.0033; B: r = −0.136, *p* < 0.05.

In contrast to S-Ca and S-Phos, PTH correlated negatively with Ca-I, given as absolute amounts or Ca-I corrected for body weight (*p* = 0.0033 and <0.05 respectively, Figure 1).

## Discussion

This retrospective study yields a highly variable intake of Ca in infants and pre-school children with CKD 3-5D. The dietary intake of Ca was below 100% (Calcium deficiency) and below 60% (severe Calcium deficiency) of the RDI in 62 and 27% of patients, respectively. If CaPB is also included in the calculation, Ca-I above the upper limit is observed in a clinically significant number of patients (~17% according to KDOQI and ~72% according to D-A-CH). On biochemical analysis, the Ca-I correlated negatively with PTH serum levels suggesting an effect on bone metabolism.

Children with a growing skeleton need a positive Ca-balance, as the Ca content of the bones increases from 25 g at birth to 1,000–1,200 mg in adulthood (16). The highest Ca requirement is during rapid growth phases (9). To meet the requirements, recommended daily intakes according to age are published in various countries. These recommendations vary in part. For the Mid-European region, the German-Austrian-Swiss (D-A-CH) reference is often used (13) and is paralleled by the recently published suggested daily Ca intake of the Pediatric renal nutrition task force (12). To our knowledge, there are no trial-based recommendations for the intake of children with CKD. Therefore, the RDI tables are used for this population and an intake of 100% of the RDI is recommended. The Ca-I is suggested to be between 100 and maximal 200% of the suggested dietary intake (SDI) in children with CKD up to a maximum of 2,500 mg by the Pediatric Renal Nutrition Task Force (12) and the KDOQI guidelines (5) respectively. The Ca-I in children with CKD was shown to decrease with progressing renal function decline (17). In line with this, we found a low dietary Ca-I in 63% of the dietary data collections and a severe Ca-I deficiency defined as an intake below 60% of the D-A-CH recommendation in 26% of children at first dietary data collection in the age group 0–6 years. These findings are in line with the reported inadequate Ca-I in an older population of children with CKD 4-5D (8). Therefore, these children are at increased risk of osseous mineralization defects. It has been demonstrated that mineralization defects were present in 29% of pediatric CKD patients with CKD 2 and up to 80% in CKD 4–5 (10). In pediatric PD patients, the incidence of mineralization defects was even higher (11). The fracture risk of pediatric patients with CKD was shown to be increased and to be associated with low cortical bone mineral density and low serum Ca (18). None of our patients had low ionized or corrected serum Ca. Serum-Ca level is not a reliable measurement of the Ca-balance, as S-Ca is maintained within the normal levels as long as possible by an increase in both, 1,25(OH)_2_ Vitamin D to increase intestinal active adsorption and PTH to increase Ca resorption from bone. In this complex interaction of these parameters of calcium homeostasis, it is essential to analyze not only serum levels of Ca but also the trend of PTH (5, 12, 19) to detect a negative Ca balance. Therefore, it was important to demonstrate, that the Ca-I correlated negatively with PTH levels in this young CKD patient population. This finding further strengthens the relevance of adequate dietary calcium intake, since the frequently administered cholecalciferol and active vitamin D metabolites increase intestinal calcium uptake, and partially compensate for the dietary calcium deficiency. High PTH serum levels, despite adequate cholecalciferol or ergocalciferol supplementation, which were shown to reduce the development of renal hyperparathyroidism in a randomized controlled trial (20) and requirement of moderate doses of active vitamin D metabolites (21), are a marker of a negative Ca balance.

Childhood CKD stage 2–5D was shown to be associated with an increased risk of fracture (18). The risk of fracture was lower in children taking phosphate binders with the majority of children taking CaPB, suggesting that the additional supplement of Ca may be protective. On the other hand, dietary Ca intake exceeding the requirements of the bone are likely to promote vascular calcifications in CKD, as shown for young adults with childhood-onset CKD and dialysis (1, 22). Extraskeletal calcifications have also been demonstrated in pediatric CKD populations (23, 24). To reduce the risk of extraosseous calcifications in CKD patients, Ca-free phosphate binders were developed and are widely used. But their use in children needs careful balance against the high Ca need of the growing bone and may result in a negative Ca balance and aggravated hyperparathyroidism. Therefore, calculation of the dietary Ca-intake will even be more important if Ca-free phosphate binders are used in children.

Adequate caloric intake is essential for the optimized growth of children with CKD. The KDOQI guidelines recommended an intake of 100% of estimated energy requirements (EER) of healthy children (5). In one study, the basal metabolic rate was similar in children with CKD compared to matched healthy children (25). In children with a GFR <75 ml/min/1,73 m^2^, an EER of 85–98% maintained acceptable growth (26). Due to the retrospective nature of the study as well as the high proportion of toddlers and infants, we were not able to calculate the EER. But the median energy intake was 120% of the recommended requirement for healthy Mid-European children [D-A-CH (13)] and thus at least adequate. However, energy intake from dialysate in peritoneal dialysis patients in the range of 4–12 kcal/kg/day depending on the peritoneal dialysis mode and dialysate glucose concentration (27, 28) was not included in our analysis, which would further increase the energy intake.

Our study has several weaknesses. First, it uses a retrospective design with the use of prescriptions or diary records. Even with general rules on when to perform dietary protocols and respective quantitative analyses, it cannot be excluded that patients with more severely compromised feeding and weight gain are overrepresented in the present study population. These patients may be more focused on using dietary counseling. This selection bias cannot be excluded in this study due to the retrospective design. The high caloric intake in our patients argues against a selection of children with poor nutrition. In addition, the length/height and weight data of our patients were within the reported range (29). Second, toddlers and infants with advanced CKD often show vomiting upon feeding. Therefore, prescribed and retained food might differ. A lower food intake than prescribed would even increase the rate of patients with Ca deficiency, whereas the number of children with an Ca-I above the suggested upper limit would be reduced and therefore may be overestimated in the present study. Third, due to the retrospective design, we could not collect 25(OH)-Vitamin D levels in all children. Therefore, the proportion of Ca absorbed in the intestine may vary extensively in patients with low or sufficient 25(OH)-Vitamin D levels (30). But low 25(OH)-Vitamin D levels would further decrease the Ca absorbed and stimulate the PTH secretion. However, most of our patients received Vitamin D supplements and active Vitamin D metabolites. Fourth, the Ca balance from dialysis was not analyzed in this study. Children can gain or lose Ca during dialysis. Most dialyzed children in the age group 0–6 years are on peritoneal dialysis. For this mode, the Ca balance was dependent on the Ca content of the dialysate, dwell time, glucose concentration, and ultrafiltration (31). The latter shows day-to-day variation, which should be included in the calculations. For exact balances, the Ca-content of the spent dialysate has to be measured. These data were not available for this study. This makes the calculation of the Ca balance difficult in an individual case, despite the fact that for total Ca balance, this is suggested to be included (12, 31).

In summary, this study shows a low calcium intake in children aged 0–6 y with CKD. If Calcium-containing phosphate binders are used, the risk of Ca overload seems to be given. Consequently, enteral Ca-I should repeatedly be monitored in children with CKD, because a significant number may otherwise be chronically under- or overexposed to enteral calcium load. The negative correlation of Ca intake with PTH suggests a significant impact on bone health in pediatric CKD.

## Data Availability Statement

The raw data supporting the conclusions of this article will be made available by the authors, without undue reservation.

## Ethics Statement

The studies involving human participants were reviewed and approved by Ethik-Kommission Fachbereich Medizin, Philipps-Universität Marburg, Germany. Written informed consent from the participants' legal guardian/next of kin was not required to participate in this study in accordance with the national legislation and the institutional requirements.

## Members of the ESPN Dialysis Working Group

Austria: C. Aufricht, Medical University of Vienna, Vienna. Belgium: J. Vande Walle, University Hospital Ghent, Department of Pediatric Nephrology/Urology, Ghent. Czech Republic: K. Vondrak, University Hospital Motol, Charles University Prague, 2nd Faculty of Medicine, Prague Finland: T. Holtta, Children's Hospital, University of Helsinki and Helsinki University Hospital, Helsinki. France: B. Ranchin, Centre de Référence des Maladies Rénales Héréditaires, Hospices Civils de Lyon and Université Lyon, Lyon. A. Zaloszyc, Hautepierre University Hospital, Strasbourg. Germany: C. P. Schmitt, University of Heidelberg, Heidelberg. G. Klaus, University Children's Hospital, Marburg. Greece: Constantinos J. Stefanidis,“Mitera” Children's Hospital. Athens, N. Printza, Aristotle University of Thessaloniki, Thessaloniki. Italy: A. Edefonti, F.Paglialonga Fondazione IRCCS Ca' Granda Ospedale Maggiore Policlinico, Milan. E. Verrina, Giannina Gaslini Children's Hospital, Dialysis Unit, Genova. E. Vidal, University-Hospital of Padova, Padova. Lithuania: A. Jankauskiene, Vilnius University, Vilnius, Lithuania. Poland: A. Zurowska, and I. Ilona Zagozdzon Medical University of Gdansk, Gdańsk Portugal: M. Do Sameiro Faria, Hospital Maria Pia, Porto. Spain: G. Ariceta, Hospital Vall d' Hebron, Barcelona. Sweden: L. Sartz, Lund University, Lasarettsgatan. Turkey: S. Bakkaloglu, Gazi University Faculty of Medicine, Ankara. A. Duzova, Hacettepe University Faculty of Medicine, Ankara. United Kingdom: D. Hothi and R. Shroff, Great Ormond Street Hospital for Children, London.

## Author Contributions

LS: design, data aquisition, data analysis, manuscript writing, and protocol development. PH: data acquisition. BT: study design, data aquisition, and medical writing. AJ, AZ, AE, and CPS: data aquisation, medical writing, and protocol development. GK: medical writing, protocol development, study design, and data analysis. All authors contributed to the article and approved the submitted version.

## Conflict of Interest

The authors declare that the research was conducted in the absence of any commercial or financial relationships that could be construed as a potential conflict of interest.
