# Supplementary material for: Enteral Ca-Intake May Be Low and Affects Serum-PTH-Levels in Pre-school Children With Chronic Kidney Disease
Source: Front Pediatr. 2021 Jul 20;9:666101. doi: 10.3389/fped.2021.666101 (PMC8329332; doi:10.3389/fped.2021.666101)
Supplement: Supplementary file 1 [file Table_1.DOC]

Recommended Calcium Intake in Infants and Preschool Children

| Age | SDI Calcium (mg) | D-A-CH Ref. Calcium (mg) |
| --- | --- | --- |
|  |  |  |
| 0 - < 4 month | 220 | 220 |
| 4 - < 12 month | 330-540 | 330 |
|  |  |  |
| 1 - <4 Years | 450-700 | 600 |
| 4 - < 7 Years | 700-1000 | 900 |

SDI: suggested intake from (1)

D-A-CH: German-Austrian-Suisse recommenfdation (2)

Literature:

1. McAlister L, Pugh P, Greenbaum L, et al. (2020)The dietary management of calcium and phosphate in children with CKD stages 2-5 and on dialysis-clinical practice recommendation from the Pediatric Renal Nutrition Taskforce. *Pediatr Nephrol*. 35(3):501-518
2. German Nutrition Society (2013) New reference values for Calcium Ann Nutr Metabol 63;186-192
