# Supplementary material for: Enteral Ca-Intake May Be Low and Affects Serum-PTH-Levels in Pre-school Children With Chronic Kidney Disease
Source: Front Pediatr. 2021 Jul 20;9:666101. doi: 10.3389/fped.2021.666101 (PMC8329332; doi:10.3389/fped.2021.666101)
Supplement: Supplementary file 2 [file Data_Sheet_1.PDF]

Date: ____ / ____ / ________

Nutrition Diary

Patient's ID:

Center ID		Patient ID		

Day of week: Mon Tues Wed Thurs Fri Sat Sun
(Please circle one)

Dietary supplements: vitamin, mineral, or herbal supplements:<br>(Please list brand, frequency, and dose)

What was your food intake for this day?

- ❑ Typical
- ❑ More than usual
- ❑ Less than usual

Time	Amount eaten or drunken (weight, volume or serving size)	Foods consumed (Please be as specific as possible)	Beverages consumed, including water (Please be as specific as possible)	Phosphate binder (type and dose)

Time	Amount eaten or drunken (weight, volume or serving size)	Foods consumed (Please be as specific as possible)	Beverages consumed, including water (Please be as specific as possible)	Phosphate binder (type and dose)

Type of water consumed: ❑ Tap water

❑ Bottled water

Bottled water brand: ______________________________

Mineral content:

Calcium ______mg/l

Sodium ______mg/l

Bicarbonate ______mg/l
