# Supplementary material for: Enteral Ca-Intake May Be Low and Affects Serum-PTH-Levels in Pre-school Children With Chronic Kidney Disease
Source: Front Pediatr. 2021 Jul 20;9:666101. doi: 10.3389/fped.2021.666101 (PMC8329332; doi:10.3389/fped.2021.666101)
Supplement: Supplementary file 3 [file Data_Sheet_2.PDF]

Date: ____ / ____ / _______

Questionnaire

Calcium intake of children with chronic kidney disease

Patient's ID: Center-ID [ ] Pat-ID [ ] [ ] [ ] [ ] [ ]

Gender: Male ❑ Female: ❑

Date of Birth: ____ / ____ / ________

Body Height: __________________ cm

Body Weight: __________________ kg

Diuresis: __________________ ml / 24h

Renal disease: ____

Dialysis: Yes ❑ No ❑

Hemodialysis: Yes ❑ No ❑

Peritoneal Dialysis: Yes ❑ No ❑

Dialysate Calcium Concentration

in mmol/l: 1,25 ❑ 1,5 ❑ 1.75 ❑ Other ❑

If other please specify: ______________________________________

Bone disease: ____

Deforming bone abnormalities:	Yes ❑	No ❑
Chronic pain related to the skeletal system:	Yes ❑	No ❑
Disabling bone abnormalities:	Yes ❑	No ❑
Aseptic bone necrosis:	Yes ❑	No ❑
Atraumatic fracture:	Yes ❑	No ❑
Renal rickets:	Yes ❑	No ❑

Nutrition

Ingestion of food:

Spontaneous	Yes ❑	No ❑
PEG	Yes ❑	No ❑
Nasogastric Tube	Yes ❑	No ❑

Nutritional Diary:

Ca-Intake	Yes	No
recorded	❑	❑

Ca-Intake	Yes	No
modified	❑	❑

Medication

Phosphate binder	Yes ❑	No ❑
- $CaCO_3$	___________ mg/d	
- CaAc	___________ mg/d	
- Ca- and Al- free	Yes ❑	No ❑
	___________ mg/d	
- Other	Yes ❑	No ❑
If other please specify:	______________________________	

	Yes ❑	No ❑
Cholecalciferol		
	___________ mg/d	___________Units/d
Other (please specify):	______________________________	

Ergocalciferol	Yes ❑	No ❑
	___________ mg/d	___________Units/d
Other (please specify):	______________________________	

Alfacalcidol	Yes ❑	No ❑
	___________ g	
Interval:	Daily ❑	Intermittent ❑

Calcitriol	Yes ❑	No ❑
	___________ g/d	
Other (please specify):	______________________________	

Cinacalcet	Yes ❑	No ❑
	___________ mg/d	
Other (please specify):	______________________________	

Recombinant Growth Hormone Therapy	Yes ❑	No ❑

Corticosteroid Therapy	Yes ❑	No ❑

Laboratory Values

		Unit	Standard Value
Albumin	______	______	______
Calcium	______	______	______
Phosphorus	______	______	______
25(OH)-Vitamin D	______	______	______
Alkaline Phosphatase	______	______	______
Parathyroid Hormone	______	______	______
Bicarbonate	______	______	______
Creatinine	______	______	______
Jaffe reaction	Yes ❑	No ❑	
Enzymatic creatinine assay	Yes ❑	No ❑	
Calcium in Urine (if available)	______	______	______
