# Supplementary material for: Enteral Ca-Intake May Be Low and Affects Serum-PTH-Levels in Pre-school Children With Chronic Kidney Disease
Source: Front Pediatr. 2021 Jul 20;9:666101. doi: 10.3389/fped.2021.666101 (PMC8329332; doi:10.3389/fped.2021.666101)
Supplement: Supplementary file 4 [file Data_Sheet_3.PDF]

# Enteral Tube Feeding

Date:

Patient's ID:

| Center ID | | Patient ID | | |
|---|---|---|---|---|
| | | | | |

| Amount | Enteral feeding / Formula type | Water supplement |
|---|---|---|
| 640 ml | | |
| 94 g | Nephea Kid | |
| 70 g | Maltocal 19 | |

| Amount | Enteral feeding / Formula type | Water supplement |
|---|---|---|
| | | |
| | | |
| | | |
| | | |
| | | |
| | | |

Water for medication administration: ________ ml

Please send us a detailed nutrition plan.

If not available please list the ingredients of the formula used:

| |
|---|
| |
| |
| |
| |
| |
